# The Taste-Masking Mechanism of Chitosan at the Molecular Level on Bitter Drugs of Alkaloids and Flavonoid Glycosides from Traditional Chinese Medicine

**DOI:** 10.3390/molecules27217455

**Published:** 2022-11-02

**Authors:** Yaqi Xu, Qianwen Sun, Wei Chen, Yanqi Han, Yue Gao, Jun Ye, Hongliang Wang, Lili Gao, Yuling Liu, Yanfang Yang

**Affiliations:** 1 State Key Laboratory of Bioactive Substance and Function of Natural Medicines, Institute of Materia Medica, Chinese Academy of Medical Sciences & Peking Union Medical College, Beijing 100050, China; 2 Beijing Key Laboratory of Drug Delivery Technology and Novel Formulation, Institute of Materia Medica, Chinese Academy of Medical Sciences & Peking Union Medical College, Beijing 100050, China

**Keywords:** berberine, phillyrin, taste masking, chitosan, hydrogen bond, molecular simulation

## Abstract

Taste masking of traditional Chinese medicines (TCMs) containing multiple bitter components remains an important challenge. In this study, berberine (BER) in alkaloids and phillyrin (PHI) in flavonoid glycosides, which are common bitter components in traditional Chinese medicines, were selected as model drugs. Chitosan (CS) was used to mask their unfriendly taste. Firstly, from the molecular level, we explained the taste-masking mechanism of CS on those two bitter components in detail. Based on those taste-masking mechanisms, the bitter taste of a mixture of BER and PHI was easily masked by CS in this work. The physicochemical characterization results showed the taste-masking compounds formed by CS with BER (named as BER/CS) and PHI (named as PHI/CS) were uneven in appearance. The drug binding efficiency of BER/CS and PHI/CS was 50.15 ± 2.63% and 67.10 ± 2.52%, respectively. The results of DSC, XRD, FTIR and molecular simulation further indicated that CS mainly masks the bitter taste by disturbing the binding site of bitter drugs and bitter receptors in the oral cavity via forming hydrogen bonds between its hydroxyl or amine groups and the nucleophilic groups of BER and PHI. The taste-masking evaluation results by the electronic tongue test confirmed the excellent taste-masking effects on alkaloids, flavonoid glycosides or a mixture of the two kinds of bitter components. The in vitro release as well as in vivo pharmacokinetic results suggested that the taste-masked compounds in this work could achieve rapid drug release in the gastric acid environment and did not influence the in vivo pharmacokinetic results of the drug. The taste-masking method in this work may have potential for the taste masking of traditional Chinese medicine compounds containing multiple bitter components.

## 1. Introduction

A bitter taste is common for most medicines, especially Chinese herbal medicines, which have been used for thousands of years through the combination of multicomponent bitter medicines for treating a variety of diseases. Usually, the quantity and variety of bitter compounds originating from pharmaceutical plants are numerous. Studies have shown that in Chinese herbal medicines, the bitterness of drugs is usually related to their drug activity and the efficacy [1,2]. However, the palatability can impact on treatment adherence, especially in pediatric medicine. For example, Erganqing Koufuye and Xiaoerganmao Koufuye, widely used in pediatrics, have good therapeutic effects, but children often refuse to take the medicines because of their poor palatability [3]. Therefore, masking the bitter taste of drugs is of great significance, especially Chinese herbal medicines containing multicomponent bitter substances.

To the best of our knowledge, commonly used taste-masking techniques include the addition of sweeteners, coatings, chemical modification, cyclodextrin inclusion, ion exchange resins, prodrugs, and microencapsulation [4,5]. However, all these methods have an unsatisfactory effect. For example, adding sweeteners is invalid for extremely bitter drugs, the film coating can only coat the tablet, which is difficult for children to swallow and difficult to divide the dose [6], and chemical modification may influence the in vivo efficacy of the drugs [7]. Overall, the biggest disadvantage of these taste-masking techniques is that they cannot mask the poor taste of traditional Chinese medicines (TCMs) with multicomponent bitter substances. It is necessary to develop a method for effective taste masking of multicomponent bitter drugs in traditional Chinese medicines.

Recently, the development of polymers such as cyclodextrin, ion exchange resin, and polyester has shown great potential for taste masking. However, these polymers are still defective in masking bitterness [8]. Li et al. [9] reported that block copolymers such as mPEG-PLLA (methoxy polyethylene glycol-poly (l-lactic acid)), mPEG-PCL (methoxy polyethylene glycol-polycaprolactone), mPEG-PLGA (methoxy polyethylene glycol-poly(lactic-co-glycolic acid)), and PEG-PLLA-PEG (polyethylene glycol-poly (l-lactic acid)-polyethylene glycol) can mask the poor taste of the drugs by forming micelles in aqueous solution to interfere with the binding between the hydrophobic groups of bitter drugs and bitter receptors in the oral cavity. This taste-masking technology may potentially improve the poor taste of oral liquids. However, the in vivo safety and solubility of these block copolymers in oral liquids need to be further optimized.

Chitosan (CS), a natural polymeric material, is widely used in pharmaceutical formulations owing to its easy availability, biocompatibility, modifiability, and in vivo degradability [10]. CS is also used for taste masking because it can form a physical barrier between the bitter drug and the bitter receptor in the oral cavity through encapsulation of the bitter substance. Furthermore, the physiochemical properties of CS make the formed microcapsules almost insoluble in the oral environment, but can rapidly dissolve and release the drug in the gastric acid environment without affecting the in vivo dissolution of the encapsulated drug [11,12]. There are many reports on CS masking the bitter taste of drugs such as enrofloxacin [13], cetirizine hydrochloride [14], ciprofloxacin [15] and ondansetron hydrochloride [16] through encapsulation. Stagner et al. [17] constructed CS nanoparticles for bitter masking of isoniazid via ionic crosslinking followed by spray drying, and inferred that besides the drug encapsulation, CS may also mask the taste through weak bonding interactions between its own amino or hydroxyl groups and those of isoniazid. In addition, CS can also achieve taste masking through electrostatic and adsorption effects [18,19,20]. Based on these reports, we speculated that the multiple taste-masking mechanism of CS may help to achieve simultaneous taste masking of multicomponent bitter substances in TCMs. To date, there are no reports on the taste masking of CS on multicomponent bitter TCMs.

The most bitter drugs in TCMs are alkaloids (e.g., berberine and quinine), terpenoids (e.g., andrographolide), and flavonoid glycosides (e.g., phillyrin and bitter amygdalin) [21,22]. Therefore, in this study, berberine (BER) from alkaloids and phillyrin (PHI) from flavonoid glycosides were selected as model drugs, and CS was used as a taste-masking material to prepare taste-masking compounds. The taste-masking mechanisms of CS on alkaloids and flavonoid glycosides at the molecular level were elaborated in detail using scanning electron microscopy (SEM), differential scanning calorimetry (DSC), X-ray diffraction (XRD), Fourier transform infrared (FTIR) analyses and computer-based molecular simulations. Moreover, based on the taste-masking mechanisms, the taste-masking technology developed in this study may simultaneously mask the poor taste of mixtures of the two drugs (BER and PHI). The taste-masking effect of CS on the bitter components of TCMs was verified through electronic tongue, in vitro drug release and in vivo pharmacokinetic analysis. The taste-masking technique developed in this study will be expected to be further extended to taste masking of TCMs with multicomponent bitter substances.

## 2. Materials and Methods

### 2.1. Materials

BER (analytical grade ≥ 98%) and clarithromycin (analytical grade ≥ 98%) were supplied by Shanghai Yuanye Bio-Technology Co., Ltd. (Shanghai, China). PHI (analytical grade ≥ 99%) was purchased from Nanjing Plant Origin Biological Co., Ltd. (Nanjing, China). Chitosan (50–90 kDa) was obtained from Sigma-Aldrich. All other chemicals and reagents used were of analytical grade.

### 2.2. Ethics Statement

All animals were handled in strict accordance with the recommendations of the Guidelines for the Care and Use of Laboratory Animals of the Ministry of Science and Technology of China. The experimental schemes and protocols were approved by the Sino Animal (Beijing, China) Science and Technology Development Co., Ltd. Ethics (approval ID: 20210154YZA-3R).

### 2.3. Animals

Specific pathogen-free Sprague Dawley rats (200 g) were purchased from SiPeiFu Biotechnology (Beijing, China). Animals were housed at temperatures of 22 ± 2 °C, humidity of 50 ± 20%, and 12 h light and 12 h dark cycles.

### 2.4. Preparation and Characterization of Taste-Masked BER/CS, PHI/CS, and BER-PHI/CS Compounds

#### 2.4.1. Preparation of BER/CS, PHI/CS, and BER-PHI/CS

**Figure 1:**
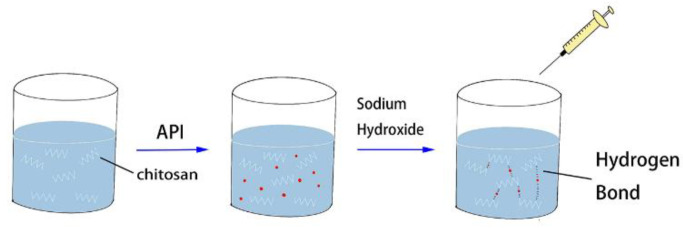
Preparation of BER/CS and PHI/CS.

As shown in Figure 1, the BER(PHI)/CS was prepared as follows: An appropriate amount of CS was dissolved in a 1% *w*/*v* acetic acid solution with continuous stirring for 30 min. The required amount (80 mg) of bitter drug (BER or PHI) was dispersed in 3 mL of CS solution with continuous stirring until well mixed. Then, the CS solution containing BER or PHI was dropped into a sodium hydroxide solution (10% *w*/*v*) with continuous stirring through a 5 mL syringe at a flow rate of 1.5 mL/min and gently stirred for 60 min to fully bind the CS and bitter drugs. The obtained precipitate was centrifuged at 2500 rpm for 5 min and dried to a constant weight in a vacuum desiccator (DZF-6050, Shanghai boxun Industry & Commerce Co., Ltd., Shanghai, China) at 25 °C for 96 h to remove the acetic acid as soon as possible. The obtained products were stored at room temperature and kept away from light. The preparation process of BER-PHI/CS was the same as above. However, the amount of BER-PHI used was 160 mg (80 mg BER and 80 mg PHI), and accordingly, the amounts of CS and sodium hydroxide were doubled.

#### 2.4.2. Binding Efficiency

BER/CS or PHI/CS containing 15 mg of BER or PHI was weighed and dissolved in 5 mL methanol. The solution was centrifuged at 4722× *g* for 10 min on a centrifuge (Sartorius AG, Weender Lanstrasse 94–108). The supernatant was collected and diluted with methanol, and the unbound drug was determined by high-performance liquid chromatography (HPLC). HPLC studies were carried out with Shimadzu Nexera XR (LC-20AD, Shimadzu, Kyoto, Japan) coupled with a UV detector. A ZORBAX C18 (250 mm × 4.6 mm, 5 µm) reverse-phase analytical column (Agilent Technologies, Santa Clara, CA, USA) was used at 30 °C. BER was detected at 345 nm and PHI was detected at 277 nm. The mobile phase was composed of 0.033 mol/L potassium dihydrogen phosphate solution and acetonitrile in 65:35% *v*/*v* for BER and purified water and acetonitrile in 75:25% *v*/*v* for PHI. The isocratic elution method used a 10 µL injection volume and a flow rate of 1.0 mL/min. BER elution time was 7.2 min and PHI was 11.33 min. A freshly prepared mobile phase was pumped through HPLC for 20 to 30 min prior to each run until a stable base line was achieved. The binding efficiency of the bitter drug was calculated using the following equation:

Binding efficiency (%) = (1 − m_1_/m_0_) × 100% (1)

where m_0_ is the input amount of BER or PHI and m_1_ is the amount of unbound free BER or PHI measured. Each sample was measured in triplicate, and the results are expressed as the mean ± standard deviation (SD).

#### 2.4.3. Morphology

The morphology of BER/CS and PHI/CS were studied using SEM (Hitachi, Tokyo, Japan). The samples were observed under an SEM microscope after gold spraying in a vacuum (10 kV, 60 s).

#### 2.4.4. Powder X-ray Diffraction (PXRD) Analysis

The PXRD patterns of PHI, BER, CS, blank CS precipitates without bitter drugs (named as blank), a physical mixture of BER (PHI) and blank with a mass ratio of 1:1 (blank:BER 1:1 or blank:PHI 1:1), BER/CS and PHI/CS were determined using an X-ray diffractometer (D8; Bruker, Bielerika, MA, USA). X-ray radiation was generated using a Cu plate with an operating voltage of 40 kV and a current of 40 mA. The samples were scanned from 5° to 50° with a scan step of 0.01° and an angular velocity of 2θ/min.

#### 2.4.5. Differential Scanning Calorimetry (DSC)

Differential scanning calorimetry (Q200; TA Instruments, New Castle, DE, USA) was used to analyze the BER, PHI, CS, blank, blank:BER 1:1, blank:PHI 1:1, BER/CS, and PHI/CS. All the samples were accurately weighed (3.00–5.00 mg), sealed in aluminum trays, and heated from 20 to 300 °C at a rate of 10 °C/min to complete the scanning analysis of the samples.

#### 2.4.6. Fourier Transform Infrared (FTIR) Spectrometry

Infrared patterns of PHI, BER, CS, blank, blank:BER 1:1, blank:PHI 1:1, BER/CS and PHI/CS were measured using a FTIR spectrometer (iS10; Nicorette, New Brunswick, NJ, USA). Approximately 2 mg of each sample was mixed with KBr at a ratio of 1:50 (sample:KBr). The mixture was pressurized to 20 MPa and then pressed into tablets and scanned using wavelengths ranging from 400 to 4000 cm^−1^. The resolution of the spectrometer was 4 cm^−1^, the signal-to-noise ratio was 50,000:1, and the number of scans was 64.

### 2.5. Molecular Docking

#### 2.5.1. Materials Studio (MS)

Materials Studio 2019 (BIOVIA, San Diego, CA, USA) was used to explore molecular simulations of the mechanism of interaction between BER, PHI, and other flavonoid glycosides (bitter amygdalin, rutin, quercetin, and baicalin) and CS. First, a CS molecule and 2–4 bitter drug molecules were used as reactants and imported into an amorphous cell for the processing of MS dynamic modeling [23]. Geometry optimization and molecular dynamics calculations (Constant-temperature, named as NPT and Canonical ensemble, named as NVT) of the formed molecules were performed to reduce the energy of the molecules and optimize the structure. Finally, the intermolecular forces were determined and calculated.

#### 2.5.2. Discovery Studio (DS)

The TAS2R 10 receptor with an extremely wide agonist range was selected as the bitter taste receptor to explore the interaction between BER, PHI, CS, and the bitter receptor using Discovery Studio 4.0 (BIOVIA, USA). Homology modeling of the TAS2R 10 receptor was performed using the I-TASSER server. All models were ranked by C-score, which is a confidence score for estimating the quality of the models predicted by I-TASSER, and the model with the highest C-score was applied as the docking receptor. Next, Discovery Studio 4.0 was used to optimize the structure and minimize the energy of bitter taste receptors and small molecules of bitter drugs and CS. Finally, docking was simulated using the C-Docker module, the binding sites and binding energies were calculated.

### 2.6. The Electronic Tongue Test

The taste-masking effect was validated using an electronic tongue system (cTongue; Shanghai Baosheng, Shanghai, China). An appropriate amount of BER, PHI, BER-PHI, BER/CS, PHI/CS and BER-PHI/CS containing the same amounts of bitter components were dispersed in 40 mL of purified water to obtain a sample solution of 1 mg/mL of bitter components and analyzed using an electronic tongue. The test was run for 120 s after the sensor reached equilibrium. The sensor was then placed in purified water and washed 1 to 6 times according to the signal curve at the time of cleaning. Each sample was measured three times in parallel.

### 2.7. In Vitro Drug Release

#### 2.7.1. Simulated Saliva

BER (5 mg), PHI (5 mg), BER/CS and PHI/CS containing 5 mg BER or PHI were dissolved in 10 mL simulated saliva (pH 6.8, composed of deionized water, NaCl, KCl, Na_2_SO_4_, NH_4_Cl, CaCl_2_·2H_2_O, NaH_2_PO_4_·2H_2_O, CN_2_H_4_O and NaF) obtained from MesGen^®^ Biotechnology (Shanghai, China) and slowly shaken at 37 °C in a shaker (120 rpm) to simulate the drug release process in the oral cavity. A sample of the solution (500 μL) was taken at 30 s and 2 min and supplemented with 500 μL of simulated saliva solution at the same temperature. The cumulative release of BER and PHI was determined using HPLC as described in Section 2.4.2. Each sample was measured in triplicate, and the results are expressed as the mean ± SD.

#### 2.7.2. Simulated Gastric Acid

Amounts of 18 mg of BER and 16.67 mg of PHI and BER/CS and PHI/CS containing 18 mg BER or 16.67 mg PHI were dispersed in 250 mL of Hydrochloric acid solution (pH 1.2, simulating the gastric acid environment, and prepared by diluting 7.65 mL of hydrochloric acid with deionized water to 1000 mL) to simulate the drug release process in gastric acid. The release of BER, PHI, BER/CS, and PHI/CS was investigated at 37 °C in a shaker (120 rpm). A volume of 1 mL of sample solution was collected at the specified time points (10 min, 30 min, 1 h, 1.5 h, 2 h, and 3 h) and supplemented with 1 mL of fresh buffer solution at the same temperature. The cumulative release of the drug was determined by HPLC as described in Section 2.4.2. Each sample was measured in triplicate, and the results are expressed as the mean ± SD.

### 2.8. In Vivo Pharmacokinetics of BER and BER/CS

Male Sprague Dawley rats (200 g) were maintained in a specific environment, at a temperature of 22 ± 2 °C and relative humidity of 50 ± 20%, for 1 week before experimentation. Before drug administration for the experiment, the rats were fasted for 12 h with free access to water. The animals were randomly divided into two groups, with nine animals in each group. The rats in the two groups were administered BER and BER/CS by gavage at a dose of 40 mg/kg. At the specified time points (0.5, 1, 1.5, 2, 4, 5, 6, 8, 12 and 24 h) after administration, blood samples (0.3 mL) were collected from the retro-orbital sinus and placed into heparinized tubes. These samples were immediately centrifuged at 4000 rpm for 10 min, and the obtained plasma was stored at −80 °C until further analysis.

A volume of 10 μL of clarithromycin internal standard working solution was added to an aliquot of 100 μL plasma. The mixture was vortexed for 10 s, followed by addition of 500 μL acetonitrile, vortexed for 1 min, and centrifuged at 14,462× *g* for 10 min. The supernatant was removed and evaporated to dryness under a stream of nitrogen. The residue was reconstituted with 100 μL acetonitrile-methanol-water (50:25:25, *v*/*v*/*v*), vortexed for 1 min, centrifuged for 10 min, and the supernatant was transferred to an autosampler for high-performance liquid chromatography–mass spectrometry (HPLC–MS) analysis. Pharmacokinetics studies were carried out with HPLC–MS (6410B, Agilent, Santa Clara, CA, USA). A ZORBAX Eclipse C18 (150 mm × 4.6 mm, 5 µm) reverse-phase analytical column (Agilent Technologies, CA, USA) was used at 25 °C. The mass spectrometer was operated in the positive ion mode using the MRM transitions at *m*/*z* 336.1 → 278.3 and *m*/*z* 336.1 → 320.2 for BER and at *m*/*z* 748.5 → 158.4 for clarithromycin (internal standard). An isocratic mobile phase of acetonitrile-10 mmol·L^−1^ ammonium acetate (containing 0.1% formic acid) (55:45, *v*/*v*) was used at a flow rate of 0.3 mL·min^−1^. The isocratic elution method used a 20 µL injection volume and BER elution time was 6.006 min. The blood concentration data of BER were fitted using the non-compartment model with DAS 3.0. The pharmacokinetic parameters were calculated using the statistical moment method.

### 2.9. Statistical Analysis

All data are shown as the mean ± standard deviation unless specified otherwise. Student’s *t*-test or one-way analysis of variance (ANOVA) was used for statistical evaluation. A * *p*-value < 0.05 was considered to be significant, and a *** *p*-value < 0.001 was considered highly significant. 

## 3. Results

### 3.1. Binding Efficiency

The precision of the HPLC method was 0.45% RSD for BER and 0.72% RSD for PHI and linearity results were R^2^ = 0.9998 for BER and R^2^ = 0.9995 for PHI, respectively. The single-factor prescription investigations of BER/CS and PHI/CS were measured using binding efficiency as an indicator, and the results are shown in Table 1 and Table 2, respectively. The results indicate that both the binding efficiency of BER/CS and PHI/CS did not increase with increasing CS dosage and the binding efficiency of PHI/CS even decreased with the CS increase. The results also showed that both the binding efficiency of BER/CS and PHI/CS increased with the increase in the amount of drug and sodium hydroxide, which is consistent with the results obtained in a previous study^5^. These results suggest that the bitter taste of the drugs could be masked by binding to the multiple binding sites of CS, and the increase in sodium hydroxide dosage could promote the binding efficiency of CS and bitter drugs. Based on the results of binding efficiency measurements, the principle of maximum drug loading and minimum amount of taste-masking materials, group 6 in Table 1 (CS, 30 mg; BER, 80 mg; and sodium hydroxide dosage of 15 mL, binding efficiency of 67.10 ± 2.52%) and group 6 in Table 2 (CS, 30 mg; PHI, 80 mg; and sodium hydroxide dosage of 15 mL, binding efficiency of 50.15 ± 2.63%) were selected as the optimal formulation for BER/CS and PHI/CS preparation.

### 3.2. Physiochemical Characterization of BER/CS and PHI/CS

**Figure 2:**
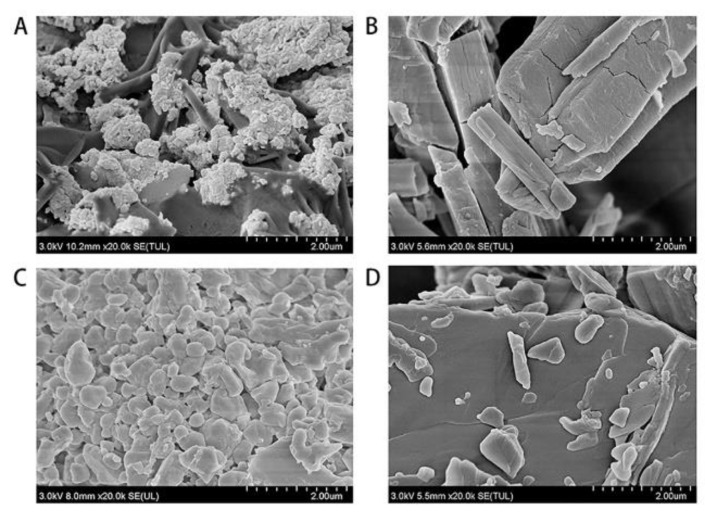
Scanning electron microscopy micrographs and particle size of BER/CS and PHI/CS. (**A**) BER/CS (measured at 2 µm scale and 20,000× magnification); (**B**) BER (measured at 2 µm scale and 20,000× magnification); (**C**) PHI/CS (measured at 2 µm scale and 20,000× magnification); (**D**) PHI (measured at 2 µm scale and 20,000× magnification).

The morphology of BER/CS and PHI/CS were determined by SEM. The SEM images (Figure 2A,B) show the morphology of BER/CS and BER at 20,000× magnification, respectively. BER/CS had multiple irregular substances with rough and uneven surfaces, which was markedly different from the morphology of BER crystals, indicating the efficiently binding of BER to CS. The morphologies of PHI/CS and PHI at 20,000× magnification are shown in Figure 2C,D, respectively. PHI/CS was amorphous and had multiple irregular sphere-like bumps but did not form regular microspheres. This was significantly different from the morphology of the PHI crystals, implying that PHI was well bound to CS. 

### 3.3. PXRD Analysis 

**Figure 3:**
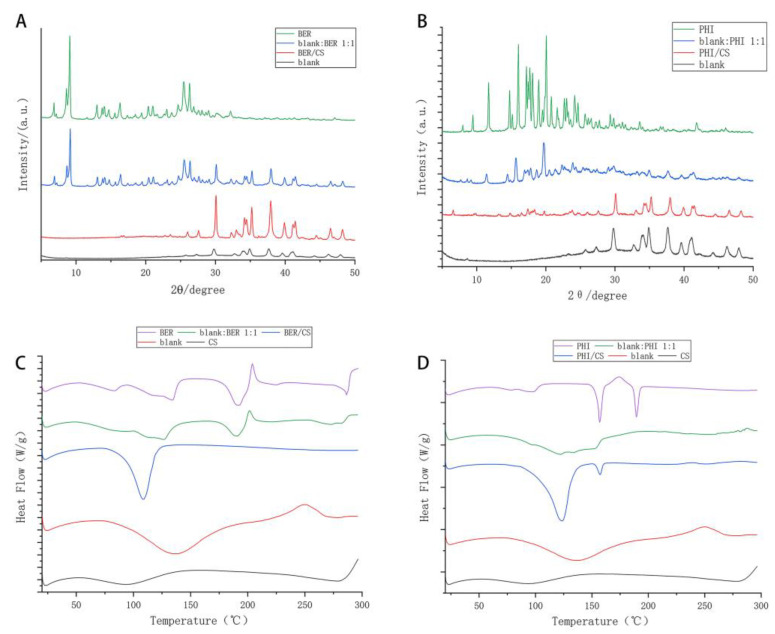
PXRD patterns and DSC patterns of BER/CS and PHI/CS. (**A**) The PXRD patterns of BER, blank CS precipitates without bitter drugs (blank), a physical mixture of BER and blank with a mass ratio of 1:1 (blank:BER 1:1), BER/CS; (**B**) PHI, blank, 1:1 blank:PHI 1:1, PHI/CS; (**C**) the DSC results of BER, blank:BER 1:1, BER/CS, blank and CS are given in this figure; and (**D**) PHI, blank:PHI 1:1, PHI/CS, blank and CS.

Crystallographic analysis of each sample was performed using PXRD, and the results are shown in Figure 3. The PXRD pattern of BER (Figure 3A) showed sharp double peaks at 8.63° and 9.12° and a series of peaks at 24.66°, 25.48°, 26.29° and 32.14°, respectively [24], which reflected the crystal structure of BER. The pattern of the blank:BER 1:1 was similar to that of the BER but showed a slight decrease in peak height. The pattern of BER/CS showed a series of sharp peaks at 30–40°, which was similar to that of the blank without bitter drugs. These results showed that the crystal morphology of BER in BER/CS was changed, indicating that BER was effectively bound to CS. 

As shown in Figure 3B, PHI exhibited a series of sharp peaks at 11.73°, 16.03°, 17.21°, 18.96°, 20.08, and 20.79°, reflecting the crystal structure of PHI. Blank showed a series of peaks at 29.75°, 34.89°, and 37.66°, respectively. The PXRD patterns of blank:PHI 1:1 were similar to those of PHI, showing a series of peaks at 10–20°, but with lower peak heights. The pattern of PHI/CS showed a series of sharp peaks at 30–40°, which is similar to that of the blank, indicating the effective binding between PHI and CS.

### 3.4. DSC Analysis

The DSC results for each sample are shown in Figure 3C,D. BER exhibited sharp endotherms at both 82.8 and 192.3 °C and a significant exothermic peak near 220 °C, as shown in Figure 2C. The exothermic peak at 220 °C was attributed to the decomposition and evaporation of BER [24,25]. The DSC thermal pattern of BER/CS showed that the endothermic and exothermic peaks of BER at 193.3 and 220 °C, respectively, almost disappeared. The result suggested that the BER of BER/CS did not melt and decompose at these two temperatures, which might be a result of the excellent binding ability of BER and CS. As shown in Figure 3D, PHI showed two sharp endothermic peaks at 156.8 and 189.4 °C, which was probably owing to the water loss from PHI and the melting of PHI, respectively. The DSC pattern of blank:PHI 1:1 retained the characteristic peaks of PHI but showed reduced peak intensity. As shown in Figure 3D, the two endothermic peaks belonging to PHI in PHI/CS shifted to lower temperatures and the endothermic peaks became broadened, less intense, and less pronounced than those of PHI, indicating that PHI was uniformly bound to CS.

### 3.5. FTIR Analysis

**Figure 4:**
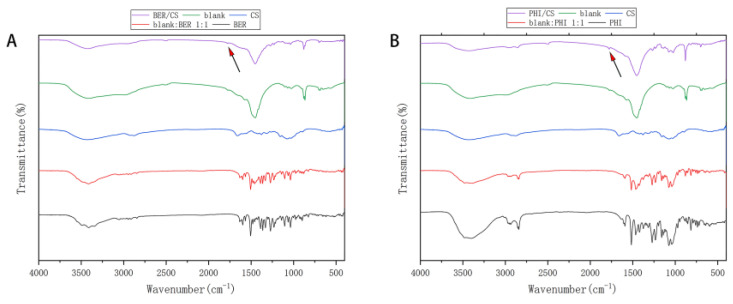
FTIR patterns of (**A**) BER/CS, blank, CS, blank:BER 1:1, BER; (**B**) PHI/CS, blank, CS, blank:PHI 1:1 and PHI.

FTIR analysis was performed to understand the molecular interaction mechanism of BER (PHI) and CS. It has been reported that nucleophilic and electrophilic groups of bitter molecules are the main groups responsible for their bitterness [26]. The FTIR spectrum of BER (Figure 4A) showed stretching vibrational peaks at approximately 3490, 3416, and 3347 cm^−1^, which belong to hydroxyl groups and a series of skeletal vibrational peaks of benzene rings at approximately 1500 cm^−1^. Figure 4A shows the FTIR spectra of blank:BER 1:1, which showed characteristic peaks similar to those of BER, consistent with previous reports [25]. In contrast, the FTIR spectrum of BER/CS was similar to that of the blank, in which the characteristic peaks belonging to BER all disappeared or decreased, but the stretching vibration peaks of hydroxyl groups shifted to higher bands (3423 cm^−1^) and the peaks broadened and spread, indicating weak interaction between BER and CS. In addition, BER/CS showed a new carbonyl peak at 1775 cm^−1^. It is speculated that the amino group of CS may interact with the hydroxyl group of the bitter molecule to form a new carbonyl group that interferes the binding of the bitter drug molecule with the taste receptor to achieve taste masking.

The FTIR spectra of PHI, CS, blank, blank:PHI 1:1, and PHI/CS are shown in Figure 4B. The characteristic peaks at 3479 and 3402 cm^−1^ are attributed to the stretching vibration peaks of the nucleophilic group of -OH in PHI. In addition, PHI had three characteristic peaks at 1604, 1592, and 1517 cm^−1^, related to its benzene ring. The FTIR spectrum of blank: PHI 1:1 showed characteristic peaks similar to those of PHI, indicating that no intermolecular interactions were generated between PHI and CS in blank: PHI 1:1. However, the FTIR spectrum of PHI/CS showed a tendency for a stretching vibrational peak originally belonging to the hydroxyl group of PHI to move to a higher band (3429 cm^−1^) with a broadening and spreading peak pattern, suggesting the same weak interaction between PHI and CS. A new carbonyl peak appearing near 1775 cm^−1^ in PHI/CS might also be related to the interaction between the amino group of CS and the hydroxyl group of PHI.

### 3.6. Molecule Docking

#### 3.6.1. MS

**Figure 5:**
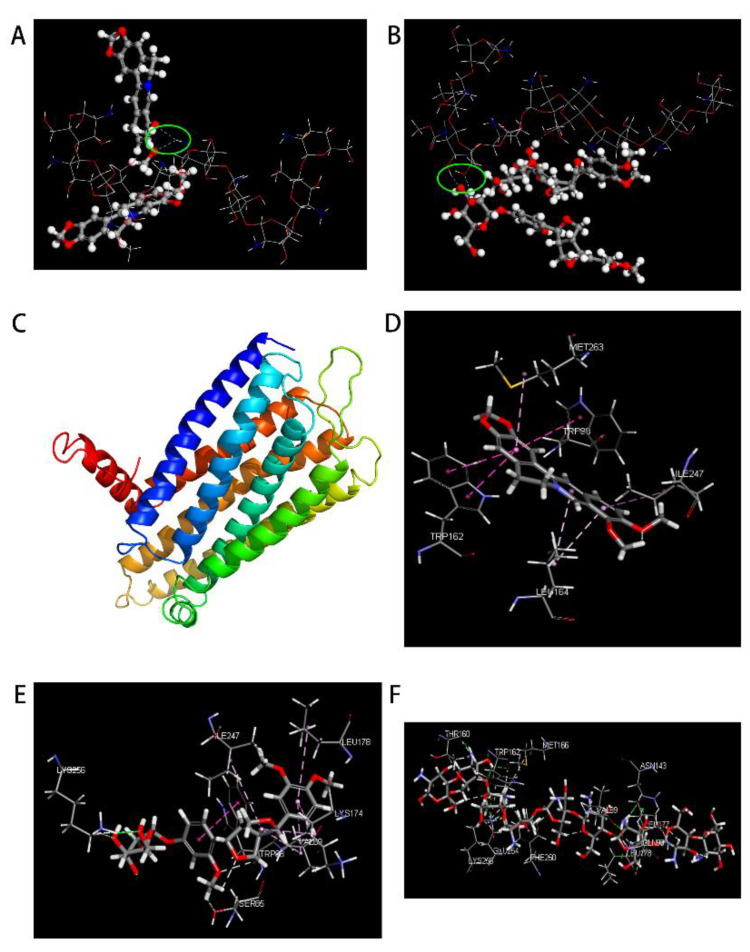
Materials Studio patterns with chitosan of (**A**) BER; (**B**) PHI; (**C**) the predicted model of TAS2R10. Discovery Studio patterns with TAS2R of (**D**) BER; (**E**) PHI; (**F**) CS.

**Figure 6:**
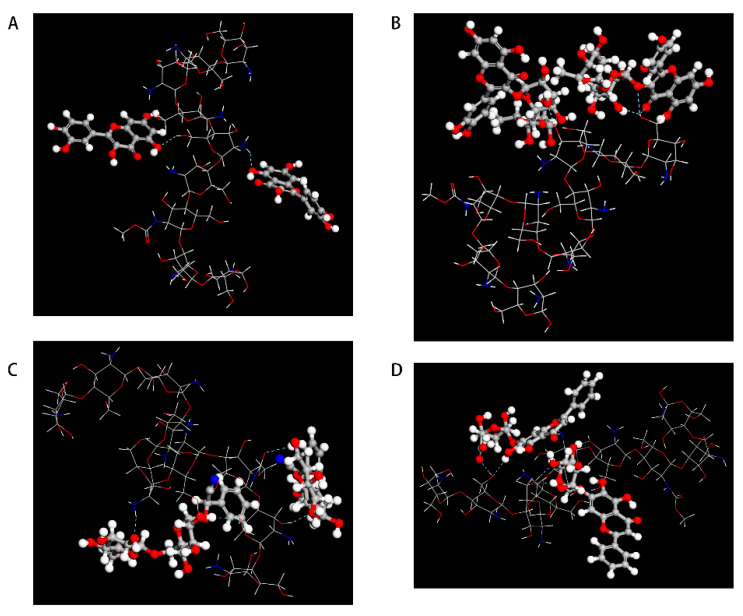
Materials Studio patterns with chitosan of (**A**) quercetin; (**B**) rutinum; (**C**) amygdalin; (**D**) baicalin.

The taste-masking mechanism of CS on bitter drug molecules was further explored using computerized molecular simulations. The results of MS analysis (Figure 5A,B) showed that CS could form hydrogen bonds between its own hydrogen and the nucleophilic group of the hydroxyl groups (-OH) of the bitter drug BER (Figure 5A) or PHI (Figure 5B) (the blue dashed line in the figure represents the hydrogen bond formed between CS and the bitter molecule). In addition, we also simulated other flavonoid glycosides, such as rutin, quercetin, baicalin, and bitter amygdalin using MS (see Figure 6). The results showed that alkaloids such as BER could bind to CS through the formation of hydrogen bonds between their own ether bonds attached to the six-membered ring and the hydrogen of CS. In contrast, flavonoid glycosides such as PHI can bind to CS by forming hydrogen bonds between their nucleophilic group of -OH on six-membered rings and the hydrogen of CS. In addition, the flavonoid glycosides could also form hydrogen bonds between the hydrogen on their own molecular structure and the hydroxyl groups of CS. 

#### 3.6.2. DS

Based on the MS results, the binding ability of BER/CS or PHI/CS on the bitter taste receptor was further simulated by DS. First, the bitter taste protein receptor TAS2R 10 model was established (Figure 5C), which belongs to the G protein-coupled receptor family. The docking results of BER, PHI and CS with the bitter taste receptor TAS2R 10 are shown in Figure 5. The bitter taste receptor binding sites of BER were MET263 and TRP88. The receptor binding sites of PHI included amino acids such as TRP88 and LYS174. The binding energies of BER, PHI, and CS to TAS2R 10 protein were calculated, and the results are listed in Table 3. We can see that the C-Docker ENERGY and C-Docker INTERACTION ENERGY of BER were −23.7863 and 30.6670, respectively. Additionally, those of PHI were −38.2429 and 49.0511, respectively. However, the values for CS were −397.9064 and 22.1711, respectively, which were significantly lower than those of BER and PHI. These results indicated that the binding ability of bitter drugs to the bitter taste receptor was stronger than that of CS, resulting in less or no bitter taste when the bitter drugs are bound to CS. This could be explained by the fact that both BER and PHI bound to the TRP88 amino acid on the bitter taste receptor TAS2R 10. However, CS does not contain this amino acid at the binding sites. Therefore, CS may mask the poor taste of bitter drugs by blocking the binding of bitter drugs to the TRP88 amino acid of the bitter taste receptor TAS2R 10.

### 3.7. The Electronic Tongue Test

**Figure 7:**
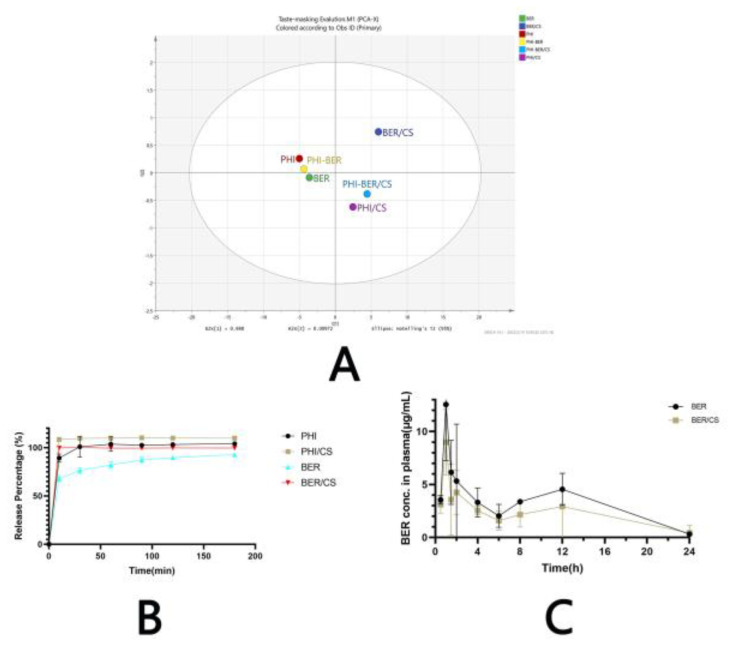
Evaluation of BER/CS, PHI/CS and BER−PHI/CS. (**A**) Electronic tongue test of BER, PHI, BER−PHI, BER/CS, PHI/CS, and BER−PHI/CS. (**B**) Dissolution profiles of BER, BER/CS, PHI and PHI/CS (*n* = 3). (**C**) BER and BER/CS blood concentration–time curve (*n* = 9).

The electronic tongue system simulates human tongue receptors through sensor arrays, while a voltametric electrochemical pulse technology simulates bitter taste signal conduction in vivo. Finally, the information is processed by an intelligent terminal to reflect bitter taste detection results. In this study, the taste-masking effect of CS on BER, PHI, and the mixture of BER and PHI (BER-PHI) was evaluated using the electronic tongue, and the results are shown in Figure 7A. The results of the electronic tongue were plotted using principal component analysis, and the Euclidean distance between the active pharmaceutical ingredient (API, including BER, PHI, or BER-PHI) and BER/CS or PHI/CS or BER-PHI/CS were calculated. The longer the Euclidean distance, the more effective taste-masking effect was achieved [27]. SIMCA 14.1 software was used for principal component analysis. The results of principal component analysis (Figure 7A) showed that compared to the API (BER, PHI, and BER-PHI), API/CS (PHI/CS, BER/CS and BER-PHI/CS) could be clearly distinguished from the API as they were distributed on the other side of the X-axis of the principal component. The results indicate the excellent taste-masking effect. Among them, the Euclidean distance of BER/CS and BER was similar to that of PHI/CS and PHI, indicating that CS has a similar taste-masking effect on these two drugs.

### 3.8. In Vitro Drug Release

#### 3.8.1. Simulated Saliva

The release of BER/CS and PHI/CS in the oral environment was investigated in simulated saliva, and the cumulative release was calculated at 30 s and 2 min, respectively. The results showed that the cumulative release of BER/CS was 9.51 ± 0.60% and 10.46 ± 0.17% at 30 s and 2 min, respectively. The cumulative release of PHI/CS was 27.48 ± 1.04% and 31.22 ± 1.66% at 30 s and 2 min, respectively. The cumulative release of BER/CS in the oral cavity was almost less than 10%, which could be considered as not inducing a significant bitter sensation in the mouth before being swallowed. The cumulative release of PHI/CS in the oral cavity at 2 min was approximately 30%, but the bitterness of PHI was relatively moderate compared to that of BER and was acceptable as a very slight bitterness. These results suggest that BER/CS and PHI/CS can mask the poor taste caused by the dissolution of bitter drugs in the oral cavity. The in vitro 30 s dissolution test in this study was conservative, because most patients swallowed the drugs much faster than 30 s. Therefore, drug release is reduced and almost no bitterness was perceived during the practical application of API/CS (BER/CS, PHI/CS, and BER-PHI/CS).

#### 3.8.2. Simulated Gastric Acid

The in vitro release of the API (BER or PHI) from API/CS (BER/CS or PHI/CS) in a simulated gastric acid environment (Figure 7B) indicated that API/CS (BER/CS or PHI/CS) could immediately release the bound bitter drug in the gastric acid environment. Both BER and PHI were released almost completely from BER/CS or PHI/CS within 10 min (BER vs. PHI: 99.73 ± 1.48% vs. 109.68 ± 1.67%). These results might result from that CS was insoluble in the oral cavity (pH 6.8) and was able to dissolve rapidly in an acidic environment at pH 1.2 in the stomach, where the large amount of H^+^ present in the acidic environment broke the hydrogen bonds formed between CS and the bitter drug, and thus releasing the drug [28]. The results from Figure 6B also display that API release is lower than API/CS—this may be because when CS is dissolved in the acid solution, it may also play a role in solubilizing drugs to some extent [29].

### 3.9. Pharmacokinetics of BER and BER/CS

The above results that the taste-masking technology not only have good taste-masking effect but also do not influence the in vivo drug release encourage us for further exploration. Herein, the in vivo pharmacokinetics are further studied. Based on the principle of ethics, only BER/CS, which showed a better taste-masking effect was chosen for further investigation. The pharmacokinetic study of BER and BER/CS was assessed in male Sprague Dawley rats following gavage administration of BER at a dose of 40 mg/kg. Firstly, the pharmacokinetic methodology was verified and the results show that the precision and linearity of the method was 9.50% RSD and R^2^ = 0.9933, respectively. The absolute recoveries, relative recoveries and matrix effects of the method were 89.336% ± 9.183%, 91.945% ± 9.421% and 1.17 ± 0.09, respectively. The mean plasma concentration–time profiles are shown in Figure 7C. The plasma concentration curves of both the BER and BER/CS groups showed double peaks, which may be owing to the hepatic–intestinal circulation of BER in vivo [30]. The corresponding pharmacokinetic parameters are listed in Table 4. The area under the curve (AUC) of the BER group was 76.897 ± 7.851 mg/L·h, whereas that of the BER/CS group was 53.847 ± 37.237 mg/L·h. The t_1/2_ of the BER and BER/CS groups was 4.667 ± 1.949 h vs. 5.127 ± 3.555 h, respectively. There were no significant differences in the AUC, t_1/2,_ C_max_ and T_max_ between the two groups. Taste masking using CS had no effect on the overall pharmacokinetics of BER.

## 4. Discussion

The palatability of drugs is very important for patients, especially the children. TCMs are often compound preparations containing multiple bitter components. Moreover, most TCMs are liquid preparations, such as traditional Chinese oral liquid medicine and Chinese decoctions. The bitter drug molecules dissolved in water can rapidly bind to the bitter taste receptors of TAS2R10 in the oral taste buds to produce bitterness [31]. Presently, although a variety of taste-masking techniques have been reported, adding sweeteners or aromatics to trigger varied sense organs for taste masking is still the main taste-masking technique for TCMs [6]. Sweeteners bind to sweet receptors in the mouth to stimulate the brain to produce sweetness perception [32]. However, after taking the bitter drug, the brain will produce sweet and bitter perceptions simultaneously, which makes the taste of the drug more complex, especially for some extremely bitter TCM components, leading to worse taste [33].

For single-component bitter drugs, taste masking at the molecular level using microencapsulation, pre-drugs and chemical modification can achieve better taste-masking effects than adding sweeteners. However, these taste-masking techniques cannot achieve taste masking for TCMs containing multiple bitter components. The interaction mechanism between the taste-masking materials and the bitter drug molecules, as well as the taste-masking mechanism, should be thoroughly investigated to achieve taste masking of TCMs at the molecular level. It has been reported that the bitter taste is mainly produced by the interaction between the nucleophilic groups (-OH, -C-O-C-, and -C=O) of bitter drugs and bitter taste receptors in the oral cavity, which stimulates a series of signal transmission from taste cells to the brain to produce taste perception [26,34,35]. Based on the bitterness formation mechanism, in the current study, it is supposed that if the taste-masking materials can block the binding of the bitter taste groups (e.g., nucleophilic groups) in drugs to the bitter taste receptors in the mouth, an excellent taste-masking effect can be realized.

**Figure 8:**
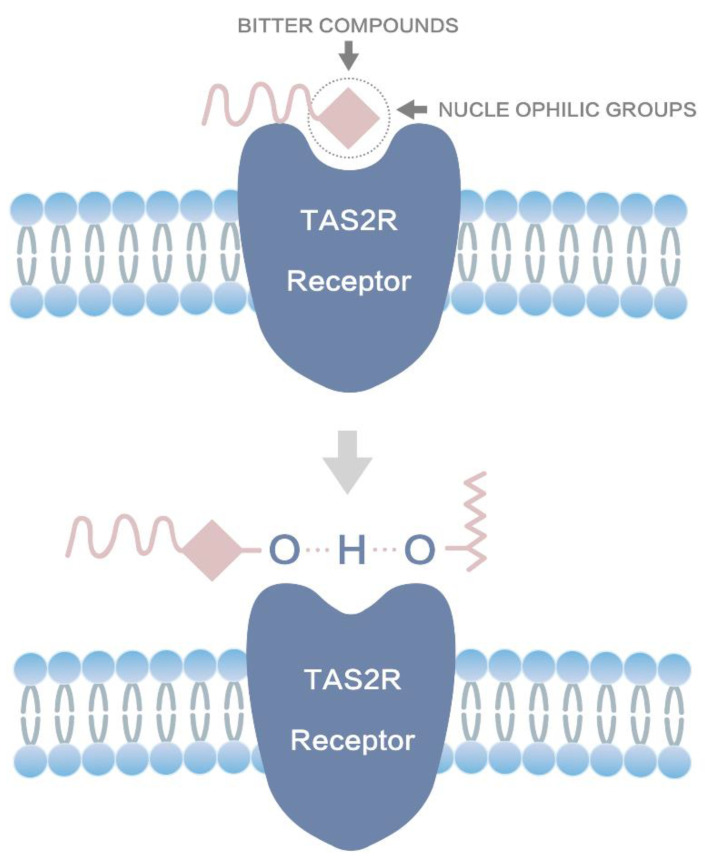
The taste-masking mechanism of chitosan with the TAS2R 10 receptor.

In this study, BER from alkaloids and PHI from flavonoid glycosides in TCMs were selected as bitter drug models. CS, a biocompatible natural material with abundant functional groups such as hydroxyl and amino groups, was chosen as the taste-masking material to mask the bitterness of TCMs at the molecular level. First, we investigated the taste-masking mechanism of CS on bitter drugs such as BER and PHI using computerized molecular simulation software (MS and DS) combined with DSC, XRD, and FTIR analyses. Based on the results, it was hypothesized that CS may improve the poor taste of BER and PHI through the following mechanisms. The primary taste-masking mechanism identified in this study (shown in Figure 8) is that CS likely forms hydrogen bonds with nucleophilic groups of bitter drugs through its own hydroxyl groups and hydrogen, thus preventing the binding of bitter drugs to the bitter taste receptors TAS2R 10, especially to the TRP88 binding site of TAS2R10 receptors to achieve the taste-masking effect. In addition, CS might mask the poor taste by partially encapsulating bitter drugs. In contrast, the results obtained also indicated that a new carbonyl group was formed between the amino group of CS and the hydroxyl group of the bitter molecule, which would prevent the hydroxyl group of the bitter drug from binding to the hydrogen-bonded taste receptor in the oral cavity.

Based on the investigated taste-masking mechanism of CS on BER and PHI, we speculated that CS might not only mask the poor taste of single drugs such as BER and PHI, but might also have a good taste-masking effect on a variety of bitter drug components or even a mixture of two or more drugs containing nucleophilic groups. The results of simulation in MS obtained in this study (Figure 6) also showed that CS could also form hydrogen bonds with other flavonoid glycosides, such as quercetin, rutinum, amygdalin and baicalin, which would hinder the binding of the drug to bitter receptors. The in vitro taste-masking evaluation using the electronic tongue also showed that CS had a good taste-masking effect on BER, PHI, and a mixture of the two (BER-PHI). The results of simulation in DS revealed that the interaction energy of CS with the bitter receptor TAS2R10 was much lower than that of the bitter drugs (BER or PHI) to the TAS2R10 receptor. All those results indicate that CS is expected to efficiently mask TCMs with multiple bitter components at the molecular level. The in vitro release and in vivo pharmacokinetic results obtained in this study further indicated that although CS had excellent taste-masking ability on the API (BER, PHI, or BER-PHI), it did not affect the API release in the stomach and showed no influence on the in vivo pharmacokinetic results of the API.

## 5. Conclusions

Overall, CS has good taste-masking ability and is not selective for bitter drugs, which makes it a safe and broad-spectrum taste-masking material. Although only BER and PHI were used as model drugs in this study, the taste-masking technology can be extended to mask the poor taste of all flavonoid glycosides and alkaloid bitter drugs. More importantly, this taste-masking technology can also be used to improve the poor taste of TCMs. The taste-masking technique in this study may provide a new idea for taste masking of TCMs, especially for some children’s TCM oral solutions, which will greatly improve the compliance of pediatric patients.

## Figures and Tables

**Table 1:** Binding efficiency of BER/CS (*n* = 3).

No.	Amount of CS (mg)	Amount of BER (mg)	NaOH Volume (10% *w*/*v*) (mL)	Binding Efficiency (%)
1	30	54	15	54.10 ± 2.98
2	40	54	15	47.99 ± 9.99
3	50	54	15	53.27 ± 6.21
4	30	20	15	51.58 ± 3.84
5	30	54	15	48.25 ± 4.39
6	30	80	15	50.15 ± 2.63
7	30	54	5	48.65 ± 7.17
8	30	54	15	56.39 ± 4.50
9	30	54	30	42.79 ± 4.16

BER and BER/CS are abbreviations of berberine and berberine/chitosan (taste-masking compounds formed by berberine and chitosan).

**Table 2:** Binding efficiency of PHI/CS (*n* = 3).

No.	Amount of CS (mg)	Amount of PHI (mg)	NaOH Volume (10% *w*/*v*) (mL)	Binding Efficiency (%)
1	30	50	15	75.96 ± 3.69
2	40	50	15	60.18 ± 1.96
3	50	50	15	48.67 ± 4.02
4	30	20	15	80.43 ± 0.33
5	30	50	15	76.03 ± 3.51
6	30	80	15	67.10 ± 2.52
7	30	50	5	61.46 ± 3.33
8	30	50	15	74.06 ± 2.31
9	30	50	30	75.56 ± 0.31

PHI and PHI/CS are abbreviations of phillyrin and phillyrin/chitosan (taste-masking compounds formed by phillyrin and chitosan).

**Table 3:** Interaction energy of TAS2R 10.

Group	C-Docker Energy	C-Docker Interaction Energy
PHI	−38.2429	49.0511
BER	−23.7863	30.6670
CS	−397.9064	22.1711

**Table 4:** Pharmacokinetic parameters of BER and BER/CS (*n* = 9).

Group	C_max_ (mg/L)	T_max_ (h)	t_1/2_ (h)	AUC (0–t) (mg/L·h)
BER	13.872 ± 3.655	1.333 ± 0.577	4.667 ± 1.949	76.897 ± 7.851
BER/CS	8.996 ± 3.086	1.000 ± 0.000	5.127 ± 3.555	53.847 ± 37.237

## Data Availability

The data presented in this study are available on request from the corresponding authors.
